# Resistance Determinants and Mobile Genetic Elements of an NDM-1-Encoding *Klebsiella pneumoniae* Strain

**DOI:** 10.1371/journal.pone.0099209

**Published:** 2014-06-06

**Authors:** Corey M. Hudson, Zachary W. Bent, Robert J. Meagher, Kelly P. Williams

**Affiliations:** 1 Department of Systems Biology, Sandia National Laboratories, Livermore, California, United States of America; 2 Department of Biotechnology and Bioengineering, Sandia National Laboratories, Livermore, California, United States of America; The University of Sydney, Australia

## Abstract

Multidrug-resistant *Enterobacteriaceae* are emerging as a serious infectious disease challenge. These strains can accumulate many antibiotic resistance genes though horizontal transfer of genetic elements, those for β-lactamases being of particular concern. Some β-lactamases are active on a broad spectrum of β-lactams including the last-resort carbapenems. The gene for the broad-spectrum and carbapenem-active metallo-β-lactamase NDM-1 is rapidly spreading. We present the complete genome of *Klebsiella pneumoniae* ATCC BAA-2146, the first U.S. isolate found to encode NDM-1, and describe its repertoire of antibiotic-resistance genes and mutations, including genes for eight β-lactamases and 15 additional antibiotic-resistance enzymes. To elucidate the evolution of this rich repertoire, the mobile elements of the genome were characterized, including four plasmids with varying degrees of conservation and mosaicism and eleven chromosomal genomic islands. One island was identified by a novel phylogenomic approach, that further indicated the *cps-lps* polysaccharide synthesis locus, where operon translocation and fusion was noted. Unique plasmid segments and mosaic junctions were identified. Plasmid-borne *bla*_CTX-M-15_ was transposed recently to the chromosome by IS*Ecp1*. None of the eleven full copies of IS*26*, the most frequent IS element in the genome, had the expected 8-bp direct repeat of the integration target sequence, suggesting that each copy underwent homologous recombination subsequent to its last transposition event. Comparative analysis likewise indicates IS*26* as a frequent recombinational junction between plasmid ancestors, and also indicates a resolvase site. In one novel use of high-throughput sequencing, homologously recombinant subpopulations of the bacterial culture were detected. In a second novel use, circular transposition intermediates were detected for the novel insertion sequence IS*Kpn21* of the IS*NCY* family, suggesting that it uses the two-step transposition mechanism of IS*3*. Robust genome-based phylogeny showed that a unified *Klebsiella* cluster contains *Enterobacter aerogenes* and *Raoultella*, suggesting the latter genus should be abandoned.

## Introduction

Carbapenems are one of few antimicrobials that have been effective against multidrug-resistant bacteria, but their utility is threatened by the emergence of carbapenem-resistant *Enterobacteriaceae* (CRE). *Klebsiella pneumoniae* is the most common CRE species in the United States, typically encountered as a hospital-acquired infection with high morbidity and mortality, and resistant to nearly all available antibiotics [1]–[4]. Enzymes that inactivate carbapenems are a major mechanism of resistance. The serine β-lactamase KPC, known since 2001, has become the most common carbapenemase in the U.S. and other countries [1]. A more recent concern is the carbapenem-active metallo-β-lactamase NDM-1, first identified in a *K. pneumoniae* isolate from 2008 [5]. Alarmingly, *bla*_NDM-1_ is often found on large conjugative plasmids along with additional antibiotic resistance determinants [6]. In some settings the gene region can form tandem repeats, elevating copy number [7]. The recent spread of *bla*_NDM-1_ both among different species and across a large geographic area has been remarkable and well documented [5]–[11].

Non-carbapenemase mechanisms of carbapenem resistance are also known. These include increasing efflux pump activity [10] and altering the profile of outer membrane porins that control access of carbapenems to the cell wall [12], [13].

*K. pneumoniae* strain ATCC BAA-2146 (Kpn2146) was the first U.S isolate found to encode NDM-1 together with a wide variety of additional antibiotic resistance determinants [14]. Susceptibility testing performed at ATCC found Kpn2146 to be resistant to every one of the 34 antimicrobial and antimicrobial/inhibitor combinations tested. While Kpn2146 resistance genes have been analyzed by both microarray [15] and (incomplete) genome sequencing [16], [17], neither approach fully elucidated the complex Kpn2146 antibiotic resistance gene repertoire. For example some Kpn2146 antibiotic resistance genes were unrecognized in the previous work, and duplicated genes were counted only once by microarray and on one contig in the incomplete genome. Even when an incomplete genome does deliver the complete gene list, the question of how a pathogen accumulates such large collections of resistance genes requires the contextual information that comes from completing the genome. The complete genome is required to reveal gene duplication events, to determine plasmid vs. chromosomal gene location, and to apply phylogenomic methods to understand the evolution of the genome. In this study, we present the completed Kpn2146 genome, identifying four plasmids, and enabling a detailed survey of its antibiotic-resistance determinants that fully explains its resistance profile. These determinants include 23 primarily plasmid-borne genes encoding antibiotic-resistance enzymes, eight of which are β-lactamase genes. It is crucial to understand how such richly endowed pathogens arise, which requires analysis of the mobile fraction of the genome. Accordingly, we surveyed genomic islands in the chromosome, mosaicism in the plasmids, and transposable elements throughout the genome.

## Materials and Methods

### DNA preparation and sequencing

*Klebsiella pneumoniae* ATCC BAA-2146 (Kpn2146) was isolated in 2010 from the urine of a U.S. hospital patient who had recently received medical care in India [14]. Genomic DNA was obtained from American Type Culture Collection (ATCC), and re-suspended in water. A previously described Illumina paired-end genomic sequence dataset from a single MiSeq run, after quality and primer sequence trimming, consisted of 3,023,757 read pairs, with reads averaging 88.3 bp [17]. A Pacific Biosciences sequence dataset (PacBio) was generated from 2 µg genomic DNA at the Yale Genome Sequencing Center, which performed the 5 kb template preparation and sequenced the library on two SMRT cells, yielding 88,073 direct reads and 1744 circular consensus sequences (size distribution: mean 2408; median 1948; range 50-18951; N50 3254 bp).

### Genome assembly

As detailed in File S1, the above MiSeq and PacBio datasets were sufficient for unambiguously assembling the complex genome with no need for additional PCR-based finishing. Novel software available at http://bioinformatics.sandia.gov/software/index.html was useful for visualizing MiSeq coverage and assembly branch points in the more challenging regions (Fig. S1 in File S1).

### Annotation

Protein-coding genes were initially identified and annotated using RAST [18], and RNA genes were annotated with careful attention to tRNA, tmRNA and rRNA genes; Rfam/Infernal [19] found 118 additional RNA genes and motifs that helped identify certain regulatory genes and sites, mobile elements, plasmid replication origins, and toxin/antitoxin systems. The Antimicrobial Resistance Database (ARDB) [20] was used to annotate antimicrobial resistance genes among the initially-called genes, testing that hits did not have better matches to other gene families; the more recently updated ResFinder [21] added only *bla*_NDM-1_ to this list of resistance genes. Explaining the Kpn2146 antibiotic resistance profile required the identification of additional genes not called by RAST. ISs were annotated using ISFinder [22]. Intact integrons were named according to INTEGRALL [23]. The chromosomal origin of replication *oriC* was identified according to [24] and PCR tests [25], [26] were adapted for *in silico* plasmid replicon-typing. Observations on a high-copy group II intron, insertion sequences, and the lack of a CRISPR system are presented in File S1.

### Phylogenetic analysis

The Kpn2146 genome was used for phylogenetic analysis, along with the 182 other *Klebsiella* reference genomes that were available at NCBI on December 20, 2013, and with five additional genomes (*Enterobacter cloacae* SCF1, *Yokenella regensburgei* ATCC 43003, *Raoultella ornithinolytica* B6 and two *Enterobacter aerogenes* genomes) included because they were originally placed in *Klebsiella*, or because a phylogenetic tree at PATRIC [27] showed that they are the closest available outgroup or fall within the *Klebsiella* clade. Multilocus sequence typing (MLST) was performed using *K. pneumoniae* data from http://www.pasteur.fr/mlst. Preliminary results showed that the 84 genomes of sequence type (ST) 258 formed a large tight clade together with the single ST512 genome; the five most divergent members of this clade were retained while the other 80 genomes of this clade were excluded from further analysis. The 108 remaining genomes were aligned into 234,232 DNA sequence blocks using default Mugsy v1.2.3 [28]. Blocks representing all ingroup genomes were selected and processed using Gblocks v0.91b [29] with the b5 = h option to remove ambiguously aligned regions, leaving 3476 blocks with a total of 2,118,733 aligned positions averaging 99.3% occupancy, which were concatenated into a supermatrix. A maximum likelihood tree was produced with RAxML v7.2.8 [30] using the GTRGAMMA substitution model. Node support values were from a bootstrap set of 150 trees produced similarly, using the fast (-x) bootstrapping function and autoFC bootstopping.

### Genomic islands

Three methods were used to find chromosomal genomic islands. i) Islander identified *att* sites for islands integrated into a tRNA/tmRNA gene [31]. ii) PHAST identified regions enriched for phage genes [32]. We also developed iii) a novel phylogenomic method termed Learned Phyloblocks (http://bioinformatics.sandia.gov/software/index.html), in which the genome is divided into regions of shared evolutionary history termed “phyloblocks”, and those phyloblocks that are “learned”, on the basis of their enrichment among the training set of Islander and PHAST islands, are used to indicate additional islands. The chromosomes of Kpn2146 and the 11 other complete reference *Enterobacter aerogenes* and *Klebsiella* genomes were aligned using mugsy. This alignment determined the “phylotype” for each position on the Kpn2146 chromosome, *i.e.*, the presence/absence pattern of the nucleotide among the reference genomes. This partitioned the Kpn2146 chromosome into phyloblock intervals defined as regions of uniform phylotype. Nonbiquitous phylotypes (those in which the sequence is not present in all 11 reference genomes) account for much (47.5%) of the Kpn2146 chromosome. This suggests that gene flux is high in *Klebsiella*, and not entirely explained by integrative genomic islands. We reasoned that some nonubiquitous phylotypes might be more indicative than others of horizontally transferred islands, if there are particularly common “highways” of island transfer among *Klebsiella* strains, as have been found in broader studies of horizontal gene transfer [33]. Phylotypes were ranked by the fraction of their nucleotides in the Islander and PHAST training islands. Phylotypes whose occurrence in training islands was >25% were termed “learned phyloblocks”, and accounted for 7.6% of the chromosome.

**Figure 1:**
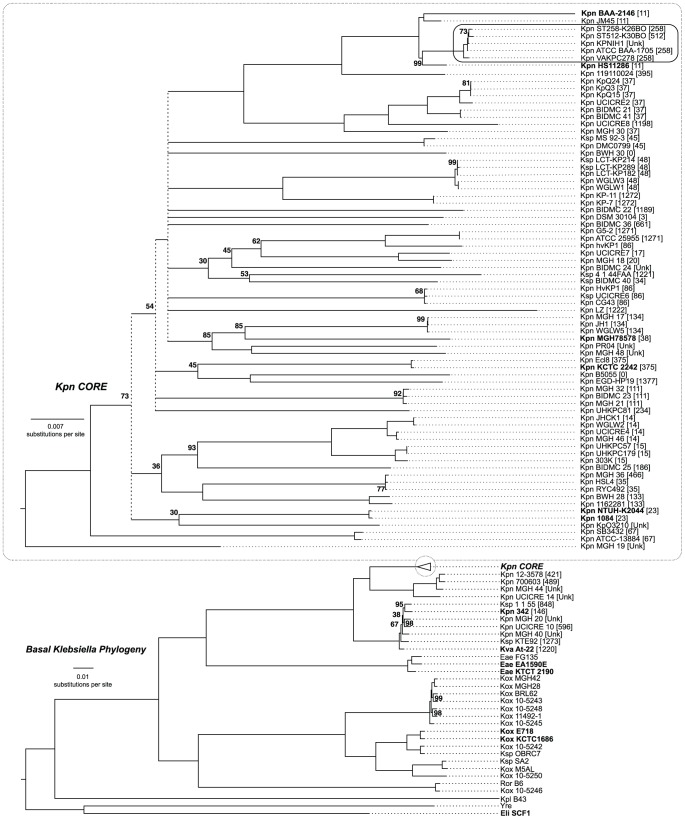
*Klebsiella* phylogeny. Tree for 108 genomes based on a 2.93-Mbp alignment, rooted at the midpoint of the outgroup (Ecl/Yre) branch. Nodes with <30% bootstrap support were combined forming the multifurcated dashed line; otherwise support values are shown only when <100%. Brackets: Kpn multilocus sequence type (ST). Inset: enlargement of the “core Kpn” phylogeny. Kpn2146 falls in a clade containing fellow ST11 strains Kpn JM45 and Kpn HS11286 and a tight clade (circled) of ST258 and ST512 strains. The ST258/ST512 clade is heavily sequenced, and represented here with only five of its most diverse members. Bold: complete genomes used for phyloblocks analysis. Species name abbreviations: Kpn, *K. pneumoniae*; Ksp, *K. sp*.; Kpl, *K. cf. planticola*; Kox, *K. oxytoca*; Kva, *K. variicola*; Eae, *Enterobacter aerogenes*; Ecl, *E. cloacae*; Ror, *Raoultella ornithinolytica*; Yre, *Yokanella regensburgei*.

Phylotypes were analyzed with Mowgli [34], parsimoniously counting gain/loss events required to reconcile our robust genome tree (Fig. 1) with its subtree of only the phylotype taxa. This allowed us to classify nonubiquitous phylotypes as either simple (explainable by a single gain/loss event), or complex (requiring multiple gains/losses). The complex class was significantly overrepresented among the learned phylotypes (36 of 38) relative to the remaining phylotypes (183 of 246) (one-sided χ^2^ test of proportions: *P*<0.005). Only two learned phylotypes were simple: Kpn2146-only and Kpn2146/KpnHS11286-only.

### PCR-based analysis of IS*Kpn21*

While abundant sequence data mapped one IS*Kpn21* copy to the chromosome and a second to pKpn2146c, less abundant sequence data suggested additional copies either in tandem repeat form or as free circles. PCR tests to distinguish these possibilities first re-examined each genomic locus. The chromosomal copy was amplified using primers C*f* (CGGTC ATAGT GTTGA TGTGGG) and C*r* (CATGT CTATT TGGTC AGAGA CGG), while the plasmid copy was amplified using P*f* (GCTTC CATGA CTGGT TGCTG) and P*r* (GATGC CAAGC CGGTA AAGTTC). Cross-copy PCRs (*i.e.*, P*f*/C*r* and C*f*/P*r*) tested for artifacts. Other primers tested for circular IS*Kpn21*: IS*f* (GCGGT TACAG GGCAT TTG) and IS*r* (GCTCT TTGAC CAGAC GATCC TG). PCR employed FailSafe enzyme mix in buffer E (Epicentre) and scheduled 2 min at 95°C, 25 cycles (15 s at 95°C, 30 s at 55°C, 3 min at 68°C), and 7 min at 68°C. Products were run on 1.2% agarose E-gels (Life Technologies).

### Plasmid mosaicism

The four plasmid sequences were queried against the July 29, 2013 *nt* database using BLASTN in default mode (*i.e.*, task “megablast”), hitting 899 complete natural plasmids. Each query and subject was self-concatenated (to avoid circular origin issues), and BLASTN was repeated, identifying regions unique to each plasmid. To define unique mosaic junctions, each query hit boundary was tested for other hits spanning the boundary (beyond 10-bp tolerance windows).

### Accession

Raw MiSeq and PacBio reads were deposited at SRA (accessions SRR931757 and SRR1185120, respectively). Genomic sequences have GenBank accessions CP006659-CP006663 and can also be browsed at http://bioinformatics.sandia.gov/klebs/.

## Results and Discussion

### Genome assembly using combined MiSeq and PacBio reads

We sequenced the genome of *Klebsiella pneumoniae* strain ATCC BAA-2146 (Kpn2146), the first U.S. isolate found to encode the NDM-1 metallo-β-lactamase. Assembly with an Illumina dataset alone was limited by poor coverage in GC-rich regions and by ambiguity at long repeats (Table S1 in File S1). However, adding a dataset of long but low accuracy PacBio reads, together with custom software for visualizing Illumina reads (Fig. S1 in File S1), allowed unambiguous assembly into five circular replicons: a chromosome and four plasmids (Table 1).

**Table 1:** Replicon copy numbers.

Replicon	Accession	Incompatibility/origin of replic.	Length (bp)	GC (%)	No. unique 21-mers^a^	Rel. mean coverage^b^	Std.Dev.
Chromosome	CP006659	*oriC*	5435369	57.29	5328730	1.00	0.63
pNDM-US	CP006661	IncA/C	140825	51.92	135388	1.65	0.67
pKpn2146c	CP006663	IncFIIA,IncFIB	117755	51.21	111911	1.77	0.93
pKpn2146b	CP006662	IncFIA,IncR	85164	52.74	67233	2.00	1.08
pKpn2146a	CP006660	ColE1	2014	49.60	1994	19.32	4.28

a Unique 21-mers were identified for each replicon, and counted among MiSeq reads.

b Mean 21-mer coverages were very similar to median values, and were normalized to that of the chromosome (which had 68.8 occurrences per 21-mer).

### Antibiotic resistance determinants

 ATCC has reported resistance of Kpn2146 to each of the 34 antimicrobial and antimicrobial/inhibitor combinations tested, including tests for 23 β-lactams (penicillins with or without inhibitors, cephalosporins, carbapenems and aztreonam), five fluoroquinolones, three aminoglycosides (tobramycin, amikacin and gentamicin), and four others (tetracycline, tigecycline, nitrofurantoin, and trimethoprim/sulfamethoxazole); see http://www.atcc.org/~/media/BA6C8F7C7C4C4649B2AEF501E51D76B8.ashx for the full list. Kpn2146 resistance genes have also been surveyed with a combination of microarray and amplicon sequencing [15]. The genome sequence fully rationalized the resistance profile, with ample evidence for one or more mechanisms explaining each observed antibiotic-resistance, and supported the gene survey. It further identified previously untested genes (like *qnrB9*), allelic multiplicity (*aac(6′)-Ib, sul1, bla*_SHV-11_ and *bla*_CTX-M-15_) and location (plasmid vs. chromosome), as well as housekeeping gene mutations (Table 2). These gene duplications can increase resistance; duplication of *bla*_SHV-11_ has been shown to increase amoxicillin-resistance 16-fold [35].

**Table 2:** Enzymes, efflux pumps, and mutations expected to confer resistance to antibiotics of clinical relevance^a^.

Enzyme^b^	Gene location(s)	Coordinates	Resistance phenotype
NDM-1 (class B)	pNDM-US Tn*125*	122191–123003	Penicillins, cephalosporins, carbapenems, inhibitor-resistant
SHV-11 (class A)^c^	1. pKpn2146b	36313–37173	Penicillins, some cephalosporins, inhibitor-sensitive
	2. Chromosome	2612996–2613856	
CTX-M-15 (class A)	1. pKpn2146b IS*Ecp1*	47130–48005	Penicillins, some cephalosporins, aztreonam,
	2. Chromosome IS*Ecp1*	5407530–5408405	inhibitor-sensitive
TEM-1 (class A)	pKpn2146b Tn*2*	50827–51687	Penicillins, some cephalosporins, inhibitor-sensitive
CMY-6 (class C)	pNDM-US IS*Ecp1*	72203–73348	Penicillins, some cephalosporins, inhibitor-resistant
OXA-1 (class D)	pKpn2146b ΔIn37	38798–39673	Penicillins, inhibitor-resistant
AAC(3)-IIe	pKpn2146b	41116–41976	Gentamicin, tobramycin, netilmicin, sisomicin
AAC(6′)-Ib (43)	pNDM-US In46	115114–115737	Tobramycin, amikacin, netilmicin, sisomicin
AAC(6′)-Ib (1)	pKpn2146b ΔInTn1331	82745–83350	Tobramycin, amikacin, netilmicin, sisomicin
AAC(6′)-Ib-cr (29)	pKpn2146b ΔIn37	38113–38712	Tobramycin, amikacin, netilmicin, sisomicin, quinolones (low-level)
ANT(3″)-Ia	Kpn23SapB In127	2297711–2298502	Streptomycin, spectinomycin
APH(3″)-Ib (StrA)	pKpn2146b IS*CR2*	53244–54047	Streptomycin
APH(6)-Id (StrB)	pKpn2146b IS*CR2*	52408–53238	Streptomycin
Sul2	pKpn2146b IS*CR2*	54108–54923	Sulfonamides
RmtC	pNDM-US IS*Ecp1*	120100–120945	Aminoglycosides (via rRNA modification)
Sul1	1. Kpn23SapB In127	2299007–2299846	Sulfonamides
	2. pNDM-US In46	116245–117084	
DfrA14	pKpn2146b In191	8281–8754	Trimethoprim
QnrB9	pKpn2146b	26074–26742	Quinolones, fluoroquinolones
Mph(A)	pKpn2146c	16503–17408	Macrolides, Erythromycin
FosA	Chromosome	667960–668379	Fosfomycin
Efflux pump	Gene Location		Probable substrate(s)^d^
AcrAB-TolC	Chromosome	1249681–1254043	Aminoglycosides, β-lactams, tigecycline, macrolides
AcrEF-TolC	Chromosome	4936203–4940465	Minor role
EefABC	Chromosome	5354323–5329922	Chloramphenicol, tetracyclines, ciprofloxacin
MacAB-TolC	Chromosome	1857393–1860445	Macrolides
MdfA	Chromosome	1781588–1782820	Aminoglycosides, fluoroquinolones, chloramphenicol
MdtG,H,K,L,M,NOP	Chromosome	e	Many possible substrates (MFS superfamily pumps)
OqxAB	Chromosome	4169609–4173960	Chloramphenicol, fluoroquinolones, trimethoprim
EmrAB	Chromosome	4218886–4221612	Nalidixic acid, hydrophobic compounds
TetA(A)	pKpn2146c Tn*1721*	19168–20367	Tetracyclines
			
Gene	Mutation		Resistance phenotype
*gyrA* Gyrase	Ser83TTC → IleATC	3763583–3766216	Quinolone, fluoroquinolones
*parC* Topo IV	Ser80AGC → IleATC	4689294–4691552	Quinolone, fluoroquinolones
*nfsA* Nitroreductase	Frameshift	1826275–1826998	Nitrofurantoin

a Excluding the resistance enzyme for bleomycin, an antibiotic used clinically only as an antitumor agent.

b Variant number from Table 1 of Ramirez *et al.* [37] is used to distinguish the AAC(6′)-Ib variants.

c Two silent differences between the two copies.

d Probable efflux substrates identified from literature sources including ARDB; the substrates list is not comprehensive and in many cases has been deduced from organisms other than *K. pneumoniae*.

e Mdt genes are scattered over the chromosome.

Eight genes for β-lactamases representing all four Ambler classes were identified; together these explain the broad β-lactam and inhibitor resistance of Kpn2146. We further identified specific resistance genes for tetracycline, trimethoprim, sulfonamides, macrolides, and multiple aminoglycoside resistance genes [36], including three *aac(6′)-Ib* variants, one shown to confer additional low-level resistance to quinolones [37] in addition to the usual spectrum of aminoglycosides inactivated by AAC(6′)-Ib which includes tobramycin, amikacin, and gentamicin C1a and C2.

The complete genome also reveals certain housekeeping gene mutations that are related to drug resistances. Its GyrA Ser83>Ile and ParC Ser80>Ile combination has previously been found in *K. pneumoniae* isolates with high-level resistance to several fluoroquinolones [38]. QnrB9 of Kpn2146, like other plasmid-encoded quinolone resistance enzymes, confers low-level resistance to fluoroquinolones, and may facilitate selection of mutations in *gyrA* and *parC* associated with high-level resistance [39]–[42]. A frameshift mutation in the nitroreductase gene *nfsA* is likely responsible for the observed resistance to nitrofurantoin [43].

The above observations explain the entire known resistance profile, except the tigecycline resistance. Mechanisms previously suggested for tigecycline resistance are mutations in the gene for the ribosomal protein S10 (Kpn2146 has the wild type allele) and mutations increasing the expression of the AcrAB/TolC efflux system [44], [45]. One mutation class causing overexpression of this efflux system is inactivation of its repressor RamR; Kpn2146 has such a *ramR* disruption (insertion of IS*Kpn18*) that can thereby explain the observed tigecycline resistance. Additional efflux systems (Table 2), such as the macrolide-specific efflux system MacAB/TolC [45], may contribute to the intrinsic spectrum of resistance, especially if overexpressed.

We also detected an early nonsense mutation that disrupts the porin gene *ompK35*, fitting with many ESBL-producing *K. pneumoniae* strains that lack OmpK35 [12]. We do not however observe the concomitant loss of OmpK36 that significantly decreases susceptibility for meropenem and several cephalosporin β-lactams; *ompK36* and *ompK37* appear to be intact [46], [47]. In a recently reported *Klebsiella* carbapenem resistance mode, the *marR* regulatory gene is inactivated and the *yedS* porin gene is active [13]; this mode is unlikely to pertain here since *marR* is intact and *yedS* is lacking in Kpn2146.

### Class 1 integrons and integron fragments

One third of the antibiotic resistance enzyme genes listed in Table 2, including all three of the *aac(6′)-Ib* alleles, are associated with five scattered class 1 integrons or integron fragments (Fig. S2 in File S1). Four of these are on plasmids, often within recognizable fragments of transposons, and the fifth is within a genomic island on the chromosome. We discuss below a case of cassette swapping where comparative analysis suggests the swap may have been mediated by homologous recombination rather than class 1 integron integrase action.

### Plasmid overview

Plasmid copy numbers were measured relative to the chromosome from the MiSeq reads, taking unique 21-mers; extremely small pKpn2146a was high-copy, while pKpn2146b, pKpn2146c and the *bla*_NDM-1_ plasmid, pNDM-US, were large and low-copy (Table 1). The large plasmids carry most of the antibiotic resistance enzyme genes in the genome (Table 2). Some mobile genes with currently unknown function may eventually prove to be new virulence or resistance genes; hypothetical genes are enriched in the two largest plasmids relative to the total genome (Table S2 in File S1).

### Conserved *bla*_NDM-1_ plasmid pNDM-US

**Figure 2:**
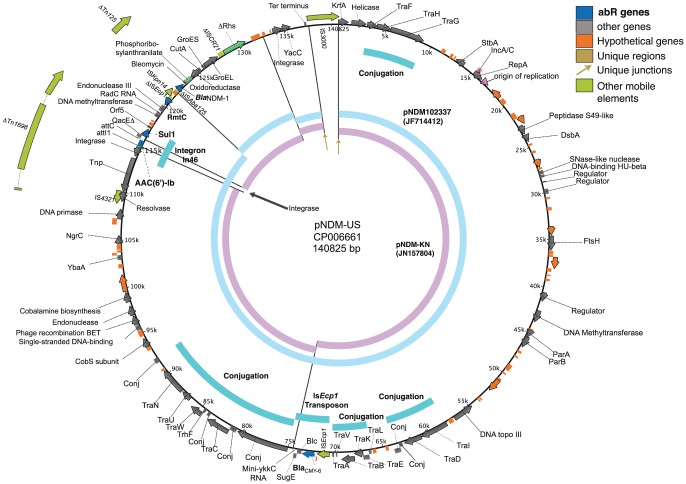
pNDM-US. Key, color coding of genes, mobile elements, and unique regions and juxtapositions, with additional colors for non-gene features. Inner ring, representative long matches to other plasmids. abR, antibiotic resistance.

The pNDM-US plasmid carrying *bla*_NDM-1_ (Fig. 2) was replicon-typed as IncA/C; it bears the IncA/C *rep* gene and iteron region, and encodes a ParAB partitioning system. Moreover, it encodes the complete set of proteins (TraABCDEFGHIKLNUVW, TrhF, DsbC, s043, s063, 123, 234, and 345) for the F-type conjugation pilus/Type IV secretion system, of the MOB_H12_ mobility class [47].

pNDM-US (140.8 kbp) is highly similar to numerous recently-sequenced plasmids, yet unique in bearing a copy of the relatively rare IS*3000* between *ter* and *krfA*. Recent insertion of IS*3000* is further supported by its 5-bp direct repeat of target sequence (DR), the first clear measurement of its DR length, in agreement with its membership in the Tn*3* family [48]. We describe the rather few differences, each discernable as distinct DNA mobility events, between pNDM-US and its two closest known relatives: pNDM-KN (JN157804: 162.7 kbp) [49] and pNDM102337 (JF714412: 166.0 kbp), which each in total share 137 kbp at >99.98% identity with pNDM-US. pNDM-KN has three large segments missing in pNDM-US: i) an IS*Ec23* insert, ii) a Tn*7*/restriction system segment, and iii) a 4-cassette integron in place of the single (*aac(6′)-Ib*) cassette integron. The second reference plasmid pNDM102337 has i) the same 1-cassette integron as pNDM-US, ii) the Tn*7*/restriction system segment of pNDM-KN and iii) bears a segment missing from both pNDM-US and pNDM-KN that carries additional resistance determinants and a full length IS*Aba125* [50].

The integron in pNDM-KN and pNDM102337 is in a fragment of Tn*1696* that has IS*4321* inserted in its remaining IR. The presence of different gene cassettes in pNDM-KN (In578), pNDM-US (In46), and other Tn*1696* variants might suggest recent integrase activity at this integron. However an alternative explanation for integron cassette swapping is by double homologous recombination in the long cassette-flanking regions that are conserved in most integrons, namely, the upstream integrase gene (5′-CS, 1352 bp) and the downstream Δ*qacE*-*sul1-orf5* unit (3′-CS, 1616 bp) [51]. This latter suggestion is supported by the presence of three of the very few point mutational differences between pNDM-US and pNDM-KN near the *att* sites in these two flanks. In the 136,910 bp shared between pNDM-US and pNDM-KN there are ten sites of small-scale indel or base-substitution; three of these are in the 5′-CS and 3′-CS, for an enrichment of (3/2968)/(7/133942) = 19.3 fold.

IS*Ecp1* has transposed into pNDM-US, bringing its 2832-bp flanking segment bearing *bla*_CMY-6_, and has been inserted intergenically into the transfer operon *tra*. The pNDM-US *bla*_NDM-1_ region is found as in pNDM-KN and in many other *Klebsiella* plasmids; its interpretation as an immobile derivative of the mobile Tn*125* of *Acinetobacter baumannii* strains has been discussed [52], [53]; here Tn*125* is truncated at one end by IS*Kpn14* and within the IS*CR21* unit at the other end.

### Mosaic plasmid pKpn2146c

**Figure 3:**
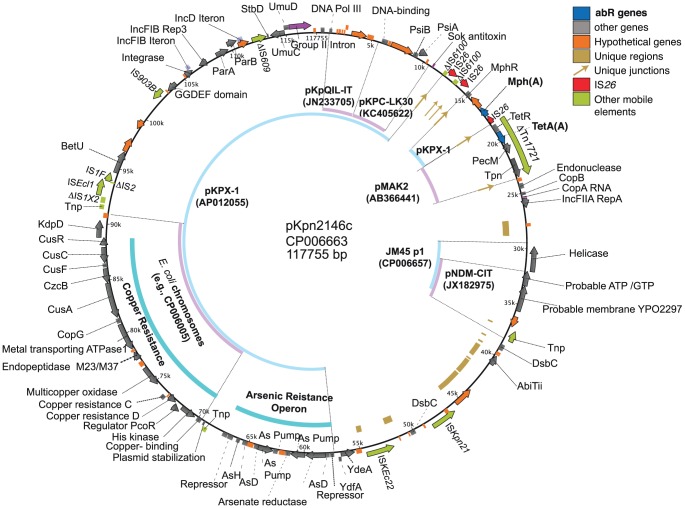
pKpn2146c. Key, color coding of genes, mobile elements, and unique regions and juxtapositions, with additional colors for non-gene features. Inner ring, representative long matches to other plasmids. Innermost black arrows, recent recombination events. HR, homologous recombination; abR, antibiotic resistance.

pKpn2146c (Fig. 3) was replicon-typed as both IncFIIA and IncFIB. It was typed to IncFIIA using the *copA* RNA gene and *copB* and *rep* protein genes, and to IncFIB through its IncFIB iteron region and *rep* gene. An iteron region IncD like that of the F plasmid was also identified.

pKpn2146c is a large mosaic plasmid, which shares much of its sequence with the *bla*_NDM-1_ containing plasmid pKPX-1, including both the large copper/arsenic resistance region and the resistance gene *mph*(A) region. pKpn2146c is also enriched for hypothetical genes (Table S2 in File S1). Three of the eleven IS*26* copies in the Kpn2146 genome occur in this plasmid (Table S3 in File S1). Directly adjacent to the *mph*(A) and IS*26* region is a Tn*1721* [54] fragment bearing the *tetA*(A) resistance gene. This transposition junction is unique among plasmids in public databases. The other end of ΔTn*1721* is truncated by an IS*26* insertion.

### Highly mosaic plasmid pKpn2146b

**Figure 4:**
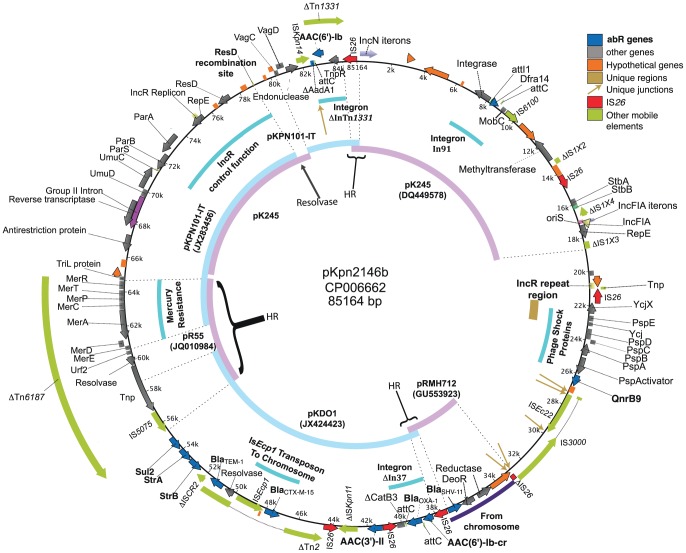
pKpn2146b. Key, color coding of genes, mobile elements, and unique regions and juxtapositions, with additional colors for non-gene features. Inner ring, representative long matches to other plasmids. Innermost black arrows, recent recombination events. HR, homologous recombination; abR, antibiotic resistance.

pKpn2146b (Fig. 4) was replicon-typed as both IncFIA (iteron unit, *oriS* and *rep* gene) and IncR. It has a largely intact IncR repeat region located 34.5 kbp apart from a locus with the *rep*, *parA* and *parB* genes and *parS* site. pKpn2146b additionally has a region of the iteron from the IncN plasmid R46 which is repeated 30.6 times, but without the IncN *rep* gene. apparently lost through IS*26* insertion followed by homologous recombination.

pKpn2146b is the richest of the plasmids in resistance determinants (12 determinants; Table 2), and the most highly mosaic, with the highest number (six) of IS*26* copies. Comparison with other plasmids shows evidence for an illegitimate recombination at the resolution site of the plasmid-encoded resolvase ResD (see Fig. 4 at coordinate 78900), where the IncR control region joins unusual sequence found elsewhere only in pK245 (DQ449578). Comparison also shows a particular pattern that we call “IS-flank switch”; one example is marked as “HR” near coordinate 38000 on Fig. 4, where homology to one reference (plasmid pRMH712) begins precisely at one end of a long repeated region (IS*26*) and extends through the IS and well into one flank, while the same pattern occurs for the other flank with a second reference (plasmid pKDO1). We hypothesize that this IS-flank switch pattern resulted from homologous recombination between IS*26*-containing parents as proposed previously [55]. This hypothesis of homologous recombination subsequent to two independent transposition events is supported by failure to find the 8-bp target sequence direct repeat (DR) expected for a recent transposition of IS26. In fact none of the six copies of IS*26* in pKpn2146b, nor any of the other five copies elsewhere in the genome, contain the DR expected for recent insertion (Table S3 in File S1), suggesting that every IS*26* copy in the genome has undergone homologous recombination more recently than transposition. We find another IS-flank switch pattern (“HR” at the top of Fig. 4), that we suspect provides an explanation of how the IncN iterons lost their associated IncN *rep* gene.

The *bla*_SHV-11_ gene originated in situ in the *K. pneumoniae* chromosome, and has been transferred to plasmids at least twice, in both cases as a chromosomal fragment flanked by directly repeated IS*26* copies [56], [57]. The pKpn2146b copy of *bla*_SHV-11_ is like the prototype in plasmid pKPN4 (CP000649), except that one of the IS*26* copies used to transmit this segment has been truncated by insertion of IS*3000*, which was then uniquely interrupted by IS*Ec22*.

pKpn2146b has much of the *bla*_TEM-1_-containing Tn*2* [58], (truncated by IS*26* at one end as found in other plasmids [55]), and further disrupted by a *bla*_CTX-M-15_/IS*Ecp1* transposition unit [59]. This pKpn2146b IS*Ecp1* copy has spawned a recent tranposition event moving *bla*_CTX-M-15_ to a chromosomal site. Chromosomal *bla*_CTX-M-15_ has not been identified in any complete genome, but has been reported at an undetermined locus in a different multilocus sequence type [60]. This recent transposition event from the plasmid used a different right end for the transposing unit (1618 bp flank) than did the earlier insertion into the plasmid Tn*2* (1315 bp flank); the resulting chromosomal copy has 100% identity with the plasmid parent and is flanked by a 5-bp DR. A partial IS*CR2* (disrupted *tnp* and *ori*) is found with its frequently associated *strA*, *strB* and *sul2* genes. The mercury-resistance operon-carrying ΔTn*6187* is only one arm of the full-length Tn*6187*, but nonetheless has the same inverted repeats at both ends as the full-length, suggesting that it alone could be a transposing element; it however lacks the expected flanking direct repeats, and thereby conforms to the IS-flank switch pattern, suggesting that its flanks may have been shuffled by homologous recombination. The integron within Tn*1331* (Fig. S2 in File S1) [61] is found truncated at one end by IS*26*, and at the other end by IS*Kpn14* leaving *aac(6′)-Ib* as its only intact resistance gene.

### Mysterious plasmid pKpn2146a

pKpn2146a (Fig. S3 in File S1) was replicon-typed as ColE, encoding RNAs I and II. The ColE1 mobilization site (*bom*) was determined by comparison to other ColE1 plasmids. The typical short ColE1 proteins that affect *ori* (Rom protein) or *bom*-site (Mob proteins) function could not be identified; indeed, none of its potentially encoded proteins show homology to any proteins in public databases. The most closely related known plasmid pB1021 (NC_019989), from *K. pneumoniae* BB1090, shares the common RNAII region and uniquely shares a second large portion of pKpn2146a. This surprisingly short (2014 bp) plasmid was supported by MiSeq coverage and verified by PCR (data not shown).

### Genomic islands determined by multiple approaches

**Figure 5:**
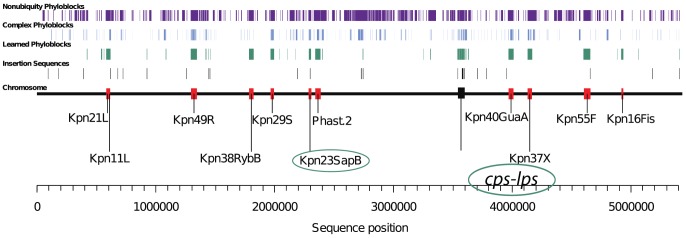
Learned phyloblocks identify a new island and the highly variable capsular polysaccharide and lipopolysaccharide synthesis gene cluster (*cps-lps*). Nonubiquity phyloblocks: those missing in at least one of the 11 reference chromosomes. Complex phyloblocks: those requiring more than one gain/loss event to reconcile the phylotype with the genome tree of Fig. 1. As a percentage of their combined 411 kbp, the learned phyloblocks mapped either to the training islands (81.9%), the two newly indicated regions (12.0%), insertion sequences (2.1%), or to small scattered regions that did not show hallmarks of islands (4.0%). Red segments: the 11 final islands (including a tandem array of Kpn21L and Kpn11L). Circles, the two newly indicated regions.

Plasmids frequently disseminate antibiotic resistance genes in *Klebsiella*, but genomic islands are also potential vehicles. Our program Islander [31] found six islands in tRNA/tmRNA genes, including a tandem island pair at a tRNA_Leu_ gene. PHAST [32] confirmed three of these and identified four additional prophage-like islands, one precisely within the gene for the short regulatory RNA RybB. The 10 resulting islands accounted for 6.3% of the Kpn2146 chromosome. We used these 10 Islander/PHAST islands (Table 3) as a training set for a phylogenomic approach to find additional islands, based on the principle that islands tend to occur sporadically among closely related strains. The Kpn2146 chromosome was partitioned into “phyloblocks”, which we define as DNA intervals where all positions share the same phylotype, *i.e.*, the same presence/absence profile among a given set of closely related genomes. We selected phyloblocks that were enriched in (*i.e.*, “learned” from) the training islands. These learned phyloblocks pointed to the island Kpn23SapB, with an integrase gene and *att* site pair, that was missed by Islander and Phast. Learned phyloblocks also pointed to the non-island genomic locus *cps-lps*, described further below. An overview of learned phyloblocks across the chromosome (Fig. 5) shows the tight mapping to *cps-lps*, mobile islands and ISs.

**Table 3:** Genomic islands.

Island	Chromosomal Coordinates^a^	Length (bp)	*att* b	Kpn HS^c^	RNA gene target	Gene content^d^	Additional support^e^	Note^f^
Kpn21L	583102–604583	21482	Yes	No	tRNA-Leu	Restriction	Islander	Tandem with Kpn11L
Kpn11L	604583–615770	11188	Yes	Yes^g^	tRNA-Leu	Phage	Islander, PHAST	Damage; Tandem with Kpn21L; Enterobacteria phage P4
Kpn49R	1296810–1345944	49135	Yes	Alt^h^	tRNA-Arg	Phage, gpII intron	Islander, PHAST	Cronobacter phage ENT47670
Kpn38RybB	1823069–1785570	37500	Yes	Yes^i^	RybB	Phage, gpII intron	PHAST	Enterobacteria phage Fels 2
Kpn29S	1966140–1994683	28544	Yes	No	tRNA-Ser	Phage	Islander	
Kpn23SapB	2286456–2309756	23301	Yes	No	No	Phage, integron, gpII intron		
Phast.2	2342484–2388101	45618	No	Yes^j^	No	Phage, gpII intron	PHAST	Salmonella phage Fels 1
Kpn40GuaA	3969748–4010194	40447	Yes	No	No	Phage, gpII intron	PHAST	Salmonella phage SPN1S
Kpn37X	4129454–4166020	36567	Yes	No	tmRNA	Phage	Islander, PHAST	Salmonella phage RE-2010; also called tmGI_Kp35 (JF764793)
Kpn55F	4603724–4658623	54900	Yes	Yes^k^	tRNA-Phe	T4SS, ParAB	Islander	Damage
Kpn16Fis	4919120–4935114	15995	Yes	Yes^l^	No	Phage	PHAST	Salmonella phage ST64B

a Islands integrated into RNA genes are assigned the orientation of the target gene; remaining islands are arbitrarily assigned the genomic orientation.

b Putative *att* sites identified at each flank.

c Status in the closely related *K. pneumoniae* HS11286 chromosome; No/Yes, absent/present in HS11286; Alt, alternative island in HS11286 at the Kpn2146 island site.

d The PHAST islands are strongly phage-like; we note where other islands have proteins suggestive of phage function, and other features of interest.

e All islands had phyloblocks support.

f Damage denotes loss of an acceptor stem nucleotide in the tRNA gene fragment [31]; phage names are the closest relatives determined by PHAST.

g CP003200.1/581777-594029; with IS*903* inserted at position 4245 with 9-bp DR; directly at tRNA-Leu *att* site with no intervening Kpn21L.

h 50403-bp alternative island at CP003200.1/1288317-1338720 with same initial 1989 bp as Kpn49R.

i CP003200.1/1778319-1808718; 2 mismatches; with 7201 bp deleted from peg.320 to 3′ end of gpII intron.

j CP003200.1/2277469-2325549; with IS*Ec22* at position 15471 with 8-bp DR.

k CP003200.1/4502805-4558770; with IS*903B* insert at position 9777 with 9-bp DR.

l CP003200.1/4819193-4835187; no mismatches.

To summarize, the 11 islands identified here (Table 3) amount to 365 kbp. Ten islands were precisely determined, having found an integrase gene and both *attL* and *attR* sites. Two islands had damage in the *attR* tRNA fragment, as has been previously observed [27]. Only five of these islands were found in the closely related strain *K. pneumoniae* HS11286.

The island Kpn23SapB has an In127 integron fragment containing an *aadA2* cassette (Fig. S2 in File S1). An upstream IS*26* insertion has displaced the integron Pc promoter, yet generated a new plausible promoter with the −35 TTGCA from IS*26*, a 17 bp spacer, and a −10 TTTCAT from the integron. This *aadA2* is the only island-borne resistance determinant identified here. However, some mobile genes with currently unknown function may eventually prove to be new virulence or resistance genes; the islands are enriched in hypothetical genes (Table S3 in File S1). Considering non-hypothetical genes, nine islands primarily possess phage genes, while Kpn55F encodes plasmid-like ParAB and some type IV secretion system functions indicative of an integrative conjugative element (ICE). Islands contain five of the six chromosomal group II intron copies.

### Operon fusion and translocation at the *cps-lps* polysaccharide synthesis locus

**Figure 6:**
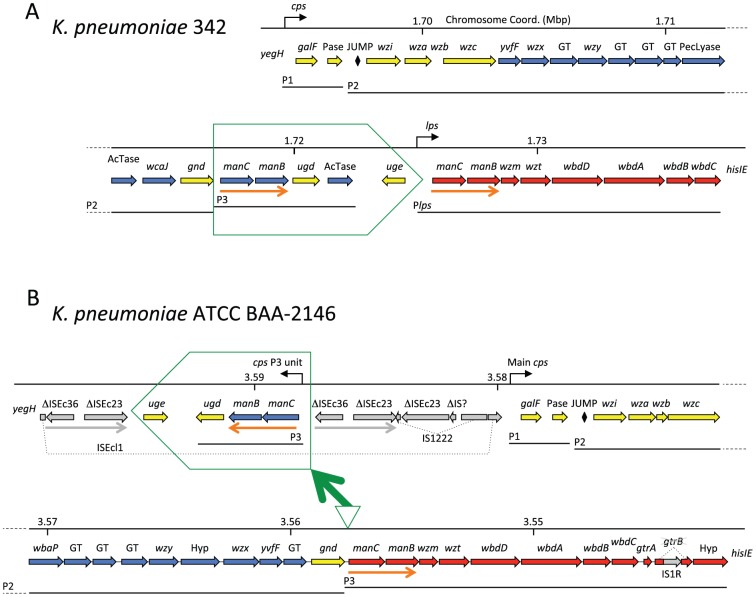
Operon translocation and fusion at the *cps*-*lps* polysaccharide synthesis locus. The *cps* P1, P2 and P3 promoters are taken from [68], while a promoter (P*lps*) has been mapped in *K. pneumoniae* MGH 78578 to the intergenic space between *uge* and the first *lps* gene [69]. **A**) The *cps-lps* region of *K. pneumoniae* 342, which is typical of *Klebsiella*. Genes of *cps* are in yellow (common in most strains) or blue (varying in gene identity, count, and order); genes of *lps* are in red. The *manCB* unit (orange arrows) is occasionally found in *cps*, and occasionally in *lps*, and here unusually in both. The diamond represents the JUMPstart DNA/RNA motif at whose *ops* sequence RfaH is loaded onto the elongating RNA polymerase in place of NusG, preventing Rho-based termination for the small number of long transcription units that are controlled by *ops*-RfaH, and physically coupling the elongating RNA polymerase to the trailing ribosome [70]. **B**) Kpn2146 *cps-lps*. The boxed *cps* P3 unit has been deleted from its usual site, and moreover translocated to a nearby position, apparently by transposition and/or homologous recombination mechanisms; note the complex pattern of surrounding IS insertions and the directly repeated flanking sequence copies (gray arrows).ΔIS, incomplete IS copy; dotted lines, gene or IS interrupted by ISs; GT, glucosyl transferase, Hyp, hypothetical.

Learned phyloblocks indicated, in addition to a new island, the genomic locus of capsular polysaccharide (*cps*) and lipopolysaccharide (*lps*) synthesis genes (Fig. 6). This region is not an integrase-mobilized genomic island, yet the *cps* cluster is known to be so highly varied as to suggest horizontal transfer of genes within the array [62]. The capsule is the outermost cell surface, a key *Klebsiella* pathogenicity determinant subject to immune surveillance. In other *Enterobacteriaceae*, the large *cps* and *lps* gene clusters are typically separate, but in *Klebsiella*, *lps* is found immediately downstream of *cps*. Nevertheless there normally appears to be transcriptional separation between *Klebsiella cps* and *lps*; *cps* terminates with the reverse-oriented gene *uge*, and an *lps* promoter has been found in the large intergenic space between *uge* and *lps* (Fig. 6A) [62]. The Kpn2146 *cps-lps* region has undergone a major rearrangement with gene-regulatory consequences (Fig. 6B). The terminal *cps* P3 transcription unit is deleted from its usual site, fusing the *lps* operon to the main *cps* operon. Morever this *cps* P3 unit has translocated to a nearby location, within a complex array of insertion sequences. In this new location the P3 unit is transcriptionally isolated, whereas at the usual location transcription could be supplemented by the upstream P2. Deletion of a polysaccharide synthesis gene cluster by homologous recombination between repeated *manCB* units has been noted before [63], but in our case the translocation has preserved the deleted *cps* subcluster.

### Circular transposition intermediates of IS*Kpn21*

Above we demonstrated transposition of *bla*_CTX-M-15_ from a resident plasmid to the chromosome by sequence comparison. Another way to assess the potential of a transposon to disseminate antibiotic resistance genes is to identify active transposition intermediates. Such intermediates have previously been found *in vivo* as free molecules unintegrated into chromosomes or plasmids, in circular, linear or tandem repeat linear forms [64], in the two-step transposition mechanism used by elements of the IS*3*, IS*30*, IS*21* and IS*256* families. We present here a novel approach for detecting circular transposition intermediates, through high-throughput sequencing. Examining the termini of IS*Kpn21*, we found MiSeq reads where IS*Kpn21* ends were linked, and separated by 5-bp direct repeat from one of the two integrated copies (Table S3 and Fig. S4 in File S1). Possible explanations for these sequences are i) that what we had assembled as single copies were instead tandem genomic repeats, or ii) that these are from circular molecules free from the genome. We tested the integrated IS*Kpn21* copies by PCR and found each to be present as a single unit, not as a tandem (Fig. S4 in File S1). We also tested for a genome-free circle (or possibly genome-free tandem) and observed the indicated PCR product. The copy number of each circle and each end of its integrated parent IS*Kpn21* copy was measured, yielding an average circle:parent ratio of 3.72%±0.84%, presuming no sequencing bias. The pKpn2146c copy of IS*Kpn21* has different direct repeat sequences at its two flanks, perhaps due to recombination between different ancestral copies. Finding only the left end direct repeat in its circle sequence suggests, without achieving statistical significance, that the left end of IS*Kpn21* preferentially attacks the right end during circularization. We propose that IS*Kpn21* and perhaps the entire IS*NCY* family use the two-step transposition mechanism of the IS*3* family.

### Using PacBio reads to detect homologously recombinant subpopulations

Above we used sequence comparison to demonstrate homologous recombination at high copy repeats as a mechanism for reassorting resistance determinants. Here we present a new method for measuring recombinant subpopulations in a bacterial culture. Small numbers of PacBio reads disagreed with the preponderant assembly pattern across the 8 copies of the rRNA operon and the 8 copies of a group II intron (Fig. S5 in File S1). To the extent that the PCR-free PacBio method is not expected or known to produce in vitro homologous recombination artifacts, our data indicated that approximately ∼4% of this bacterial culture was recombinant across these repeats.

### *Klebsiella* phylogeny revises taxonomy

We expanded the phylogenetic analysis used in our learned phyloblocks analysis, to produce a robust genome-based phylogenetic analysis of *Klebsiella* (Fig. 1). This reveals a clade with Kpn2146 and fellow members of multi-locus sequence type (ST) 11, *K. pneumoniae* HS11286 and *K. pneumoniae* JM45, from which sprang a tight clade of heavily sequenced *K. pneumoniae* ST258 and ST512 hospital strains; Kpn2146 is the only *bla*_NDM-1_-containing member of this clade (or indeed our entire tree). The surrounding and subtending of *Enterobacter aerogenes* and *Raoultella* with *Klebsiella* taxa of long standing, with 100% bootstrap support, suggests that all should be subsumed under *Klebsiella* and that the genus *Raoultella*, defined based on analysis of only two genes [62], should be abandoned.

## Conclusions

A single relatively small Illumina read set, combined with a PacBio set of longer but less accurate reads, was sufficient to assemble the genome despite the numerous repeat and high-GC regions, with no need for gap closure by PCR. Moreover we demonstrated direct detection of an active transposable element by high-throughput sequencing. Our novel read-visualization tools (http://bioinformatics.sandia.gov/software/index.html) were useful for working through problematic areas, and this software was developed into a greedy contig assembler.

The known extensive antibiotic-resistance profile of *Klebsiella pneumoniae* ATCC BAA-2146 (Kpn2146) was explained and additional resistances, which remain to be tested experimentally, were suggested by the genome sequence. Several mechanisms were identified for the mobility of resistance genes: i) acquisition of plasmids and genomic islands, ii) integron cassette swapping (whole or partial integrons account for eight antibiotic-resistance genes), iii) transposition events from chromosome to plasmid leading to greater disseminability of resistance, and vice versa leading to greater stability in the genome, and iv) homologous recombination at high copy repeats. Gaining more insight into such key evolutionary mechanisms, beyond simply identifying them, often comes through technological advances. Here we have made novel use of high-throughput sequencing technologies to inform both transposition and homologous recombination.

Numerous mobile genetic elements were identified. The eleven genomic islands were identified by three different methods that were based on the preference of islands for tRNA gene integration sites [31], clustering of phage genes [32], and a novel phylogenomic approach introducing phyloblocks, DNA segments with shared phylogenetic profiles that may be applicable in more general studies of horizontal gene transfer (Fig. 6). A recent study of the closely related ST258 *K*. *pneumoniae*, published while our manuscript was under review, also found numerous islands and indicated the *cps* locus as the major non-island chromosomal site of variation among strains [65].

The Kpn2146 genome illustrates the massive arsenal of antibiotic-resistance genes, and agile repertoire of mobile genetic elements, that the emerging CRE bacteria have at their disposal for adapting to new challenges. Homologous recombination at multicopy sequences [66], site-specific recombination by resolvases [67], switching of integron cassettes, and transpositions have shaped *Klebsiella* plasmid mosaicism.

## Supporting Information

File S1 **Supplementary materials: additional genomic features and supplementary tables, figures, and references.**
(DOCX)
