# Supplementary material for: Resistance Determinants and Mobile Genetic Elements of an NDM-1-Encoding Klebsiella pneumoniae Strain
Source: PLoS One. 2014 Jun 6;9(6):e99209. doi: 10.1371/journal.pone.0099209 (PMC4048246; doi:10.1371/journal.pone.0099209)
Supplement: File S1 — Supplementary materials: additional genomic features and supplementary tables, figures, and references. (DOCX) [file pone.0099209.s001.docx]

**FILE S1. SUPPLEMENTARY MATERIALS**

for “Resistance determinants and mobile genetic elements of an NDM-1-encoding *Klebsiella pneumoniae* strain”

by C.M. Hudson, Z.W. Bent, R.J. Meagher, and K.P. Williams

I. Additional genomic features 1

Assembly details 1

Mobile group II intron 2

Insertion sequences 3

Lack of CRISPRs 3

II. Supplementary tables 4

1. MiSeq coverage of contig joint classes inversely correlated with GC content 4

2. Enrichment of hypothetical genes in mobile genetic elements 4

3. Direct repeat (DR) of target sequence for full-length transposable elements 5

III. Supplementary figures 6

1. Novel software applied in visualizing the last gap in the genome assembly 6

2. Scattered class 1 integron and integron-derived regions 7

3. Plasmid pKpn2146a 8

4. Free IS*Kpn21* circles 9

5. Support for rRNA operon assembly 10

6. S.ma.I1 group II intron preference for promoters 11

IV. Supplementary references 12

**I. ADDITIONAL GENOMIC FEATURES**

**Assembly details.** The genome was assembled using existing assembly tools and new scripts available at <http://bioinformatics.sandia.gov/software/index.html>. The MiSeq dataset was assembled using the runAssembly wrapper of the GS De Novo Assembler v2.8 with default settings, yielding 712 contigs longer than 500 bp, of mean length 7877 bp. The perl script readStepper was developed to extend an input genomic seed sequence (typically ~20 bp taken from near the contig end of interest). The algorithm collects matching reads, orients them appropriately, trims off the seed and upstream sequence, sorts remaining sequences alphabetically, and prints them aligned without gaps from the point of seed trimming, producing a visual display for distinguishing the flanks of multi-copy seeds (Fig. S1 in File S1). A companion script mateStepper collects the mates of the seed-hitting reads, whose alignment reveals longer extensions from the seed. The above tools fully delimited the small plasmid pKpn2146a and refined the contig set by clarifying difficult repetitive regions: the internal spacers and flanking sequences of the eight rRNA operon copies, the flanks of the eight copies of a long group II intron, and the five class 1 integron-derived regions.

The script deNovo iterates the above collect/trim/sort strategy to produce an independent de novo assembly. A pre-built index (prepared by indexReads) records for each unique 21-mer found in all the reads, the “fate” of extension (whether blocked by low coverage or by exiting a duplicated sequence, or extended without blockage) and all branchings that follow a contig terminus, providing contig-extension information not available for the GS contigs. This script did not resolve redundancies among the output contigs, but did provide support for several junctions between GS contigs.

The genome was closed using the PacBio sequencing dataset. Direct and consensus PacBio reads were merged and subjected to BLASTN analysis against the GS contigs, identifying candidate bridges that would join the ends of at least one contig pair, recording the match extent, distal to the junctions for each partner contig. This produced a list of unambiguous junctions, and ambiguities caused by repetitive contigs that were resolved by individual studies of each repeat type; Fig. S5 in File S1shows such analysis of the most difficult repeat type, the rRNA operon. Many of these junctions were gaps that could not be filled using our extended MiSeq contigs. To fill such gaps, the PacBio data were not sufficiently reliable; they were filled comparatively, by aligning relevant PacBio reads, taking their consensus for the gap sequence, flanking it with 100 bp from the GS contig pair, and querying the tentative junction sequence against GenBank. In every case, a reference sequence could be found that matched nearly perfectly the GS contig flanks and matched well the PacBio consensus gap. The retrieved reference sequence was added to the PacBio alignment, and obvious mismatches in the gap were corrected toward the PacBio consensus. The 368 gaps filled this way totalled 68467 bp. Small numbers of PacBio reads suggest recombination among the eight copies of the rRNA operon (Fig. S5 in File S1) and among the eight copies of a group II intron, which may reflect in vivo recombinant subpopulations in the Kpn2146 culture.

After PacBio-based joining, three plasmid circles were completed but three linear (unclosed) genomic pieces remained: a long plasmid contig, a long chromosomal contig, and an unincorporated original GS contig; *i.e.*, three closing junctions lacked PacBio data. Use of readStepper defined these junctions, circularizing the chromosome and incorporating the last GS contig into the last plasmid contig (Fig. S1 in File S1).

A final review revisited the MiSeq data (with read/mateStepper) and the PacBio data. Each copy of multicopy GS contigs, except those with eight or more copies (16S and 23S rRNA genes, the group II intron, and IS*26*), were manually reconstructed from MiSeq data. Each discrepancy with the reference plasmid pNDM-KN [[1](#_ENREF_1)] or with an independent incomplete Kpn2146 genome sequence [[2](#_ENREF_2)] was reinvestigated. This did not alter contig order, but did correct base substitutions and small indel errors altering 21 protein sequences, with six reunifications of frameshift-split CDSs.

Retrospective analysis of contig junctions shows that gaps that could be filled with the extended MiSeq contigs had moderate GC content, while gaps that could not be filled this way had substantially elevated GC content and reduction in MiSeq read coverage (Table S1 in File S1). Likewise low MiSeq coverage of GC-rich regions prevented closure of junctions even where the initial contigs overlapped or abutted.

**Mobile group II intron.** The group II intron S.ma.I1 [[3](#_ENREF_3)] was present in eight copies, bearing the reverse transcriptase gene that confers mobility. In all but one case, a model for the uninterrupted target site could be found at GenBank. Five of the eight copies are 7 bp downstream from a predicted transcription start site and upstream of a ribosome-binding site that imposes a strong apparent integration site preference (Fig. S6 in File S1). The mobile intron is itself carried within mobile elements, either in chromosomal genomic islands or on plasmids. Even for chromosomal copy B that does not appear in our list of genomic islands, the functions of neighboring genes suggests that it may be part of a yet unrecognized island.

**Insertion sequences**. The most abundant IS is IS*26*, with 11 full copies. Other multicopy ISs were IS*Kpn1* (5 copies), IS*Ecp1* (4 copies, each having transposed with right-flanking sequence), and IS*Ec22*, IS*Kpn14*, IS*Kpn18*, IS*3000* and IS*6100* (2 copies each). A novel IS (IS*Kpn21*) was found in two copies, allowing identification of its inverted repeat terminal sequences and its target sequence direct repeat (DR) length as 5 bp; its transposase places it in the IS*NCY* family. Table S3 in File S1 shows DRs for complete ISs with terminal inverted repeats; strikingly, none of the IS*26* copies has the expected 8-bp DR. One copy of IS*3000* suggests that the previously undetermined IS*3000* DR length is 5 bp. Numerous additional ISs were found in incomplete forms. Transposase genes were freshly annotated using the several pfam HMMs for transposases. Most of these mapped to those partial or complete ISs that had been identified at the nucleotide sequence level. Two more transposase genes could be identified as the rolling-circle replicating IS*CR*s that can mobilize neighboring genes, however the IS*CR2* and IS*CR21* copies found here are truncated, each missing its origin of replication. An additional 14 transposase genes remained unassigned; all had BLASTP matches to transposases in the ISFinder database [[4](#_ENREF_4)], but none of the corresponding ISs could be identified at the nucleotide sequence level. These were annotated as insertion sequence fragments, although some of them may be yet unidentified intact ISs. These isolated transposase genes were significantly enriched in the chromosome (10 of 14) relative to those assigned to ISs or transposons (27 of 69) (one-sided χ^^ test of proportions: *P=*0.027), suggesting that the chromosome retains more degenerate ISs than do the plasmids.

**Lack of CRISPRs**. The CRISPRfinder service (http://crispr.u-psud.fr) found two single-spacer CRISPRs, but flagged them as questionable. These were then ruled out because they are both ubiquitous among the complete *Klebsiella* genomes, and none of the major CRISPR-associated proteins Cas1, Cas2, Cas3, Cse5e, nor Cse3 (present in some *Klebsiella* genomes) could be found encoded in Kpn2146.

**II. SUPPLEMENTARY TABLES**

TABLE S1. MiSeq coverage of contig joint classes inversely correlated with GC content

| Sequence class^a^ | | No. | | Mean length (bp) | | GC content (%) | |
| --- | --- | --- | --- | --- | --- | --- | --- |
| Overlap/abutment joint | 320 | | 116.1 | | 65.8 | |  |
| MiSeq-fillable gap joint | 80 | | 294.1 | | 51.2 | |  |
| Non-MiSeq gap joint | 368 | | 286.1 | | 67.9 | |  |
| Non-joint contig-internal | 768 | | 7309.8 | | 56.3 | |  |

^a^ The overlap or gap sequence for each of the 768 joints was taken retrospectively, adding 50 bp from both flanking contig sequences. Joints were classified into three types:

“Overlap/abutment”, Newbler failure due to poor MiSeq coverage but leaving no gap; “MiSeq-fillable gap”, Newbler failure despite good MiSeq coverage;

“Non-MiSeq gap”, poor MiSeq coverage left gap that required additional data (PacBio and comparative) to fill.

The remaining non-joint sequences provide a GC content baseline.

TABLE S2. Enrichment of hypothetical genes in mobile genetic elements

| Genome fraction | No. CDS | | No. hypoth. | | Hypoth. (%) | | *P*-value^a^ |  |
| --- | --- | --- | --- | --- | --- | --- | --- | --- |
| Total | 5640 | 989 | | 17.5 | | --- | | |
| Chromosome | 5310 | 888 | | 16.7 | | NS | | |
| All Plasmids | 330 | 101 | | 30.6 | | 1.8e-9* | | |
| pNDM-US | 124 | 53 | | 42.7 | | 1.9e-11* | | |
| pKpn2146c | 114 | 31 | | 27.2 | | 5.3e-3* | | |
| pKpn2146b | 90 | 15 | | 16.7 | | NS | | |
| pKpn2146a | 2 | 2 | | 100.0 | | 3.4e-2 | | |
| All Islands | 426 | 208 | | 48.8 | | 4.6e-55* | | |
| Kpn21L | 16 | 10 | | 62.5 | | 7.6e-6* | | |
| Kpn11L | 12 | 4 | | 33.3 | | NS | | |
| Kpn49R | 56 | 34 | | 60.7 | | 1.2e-16* | | |
| Kpn38RybB | 47 | 13 | | 27.7 | | 2.6e-2 | | |
| Kpn29S | 32 | 24 | | 75.0 | | 9.2e-17* | | |
| Kpn23SapB | 27 | 8 | | 29.6 | | NS | | |
| Kpn40GuaA | 51 | 31 | | 60.8 | | 9.0e-8* | | |
| Kpn37X | 45 | 12 | | 26.7 | | 4.1e-2 | | |
| Kpn55F | 59 | 35 | | 59.3 | | 1.9e-16* | | |
| Kpn16Fis | 22 | 13 | | 59.1 | | 1.0e-6* | | |
| Phast.2 | 59 | 24 | | 40.7 | | 6.2e-6* | | |

^a^ Significance of the hypothetical gene fraction was evaluated with the one-sided χ^2^ test of proportions relative to the total genome. *P*-value is shown when significant, and asterisked when <0.001. NS, not significant.

TABLE S3. Direct repeat (DR) of target sequence for full-length transposable elements

Left…right flanks of IS/Tn^a^ DR^b^ Locus

IS*26* (IS*6* family), expected DR length = 8 bp

tgaaaaccgccactgGGCAC…GTGCCctggccgatgaagtt No chromosome/2297549-2296730

catagtcgagattggGGCAC…GTGCCggttttcgaggatat No chromosome/2301859-2302678

tggcaggcttttcccGGCAC…GTGCCcgccgagctatggtg No pKpn2146c/12327-13146

ccgtgctcgtgctgcGGCAC…GTGCCggagtgattcactgt No pKpn2146c/13688-14507

ctacgggctttttcaGGCAC…GTGCCggattgaatataacc No pKpn2146c/18297-17478

gtagttaattttttgGGCAC…GTGCCcgctcagctggttgg No pKpn2146b/14157-14976

ttgctgccgcctggaGGCAC…GTGCCgaaaattgcccgtac No pKpn2146b/21817-20998

gaataacccggcgttGGCAC…GTGCCtttaagcgtgcataa No pKpn2146b/37247-38066

ctatacctatcgagaGGCAC…GTGCCccacatcttttgtca No pKpn2146b/41072-40253

atccccgaaaaatagGGCAC…GTGCCaatgtgggcgtctgg No pKpn2146b/44492-43673

tattgagaacaaaaaGGCAC…GTGCCaaaatatcgtgccag No pKpn2146b/85163-84344

IS*6100* (IS*6* family), expected DR length = 8 bp

ctcaatactcgtgtgGGCTC…GAGCCtccgtcgccatgctc No^c^ chromosome/2300920-2301799

ctcaatactcgtgtgGGCTC…GAGCCtccgtcgccatgctc No^c^ pKpn2146b/9323-10202

IS*Kpn1* (IS*3* family), expected DR length = 3-4 bp

gcccgcgcaagcgtaTGGAC…GTCCAgtagcgccgccgggc Yes chromosome/393395-391951

gcccggtaagcgcagTGGAC…GTCCAgcagcgccaccgggc Yes chromosome/1456495-1457939

gcccgcgcaagcgcaTGGAC…GTCCAgcagcgccgccgggc Yes chromosome/2191732-2193176

gcccggctgcgctgcTGGAC…GTCCAtgcgcttgcgcgggc Yes chromosome/3707551-3706107

gcccgcgcaagcgcaTGGAC…GTCCAgcagcgccgccgggc Yes chromosome/5178661-5177217

IS*Kpn18* (IS*3* family), expected DR length = 3 bp

gcgcttaacgctgacTGTTG…CAACAgacaaacagccgctg Yes chromosome/925511-924209

aatcgcgaggtacagTGTTG…CAACAcagctcgttgagcaa Yes chromosome/1445375-1446677

IS*Ecl1* (IS*3* family), expected DR length = 15-16 bp

ggccaacgcccataaTGATG…GATCActagactggccccct No pKpn2146c/92285-93620

IS*1F*, IS*1R*, IS*1X4* (IS*1* family), expected DR length = 9 bp

gagaatgtcggggatGGTGA…TTACCgtcggggatttccgc Yes chromosome/3544726-3543959

ggagaacagatgattGGTAA…TTACCgaatatcggatgatt No pKpn2146c/93719-94486

ttctgtccagttctgGGTAA…TTACCcctcaatcaaacgtg No pKpn2146b/16795-16258

IS*Kpn14* (IS*1* family), expected DR length uncertain

ataccagacatttttGGTGA…TTACCcaagttgccatgtca No pNDM-US/121200-121967

tcagtttggcgtgcgGGTGA…TTACCctcgccgcgttgttt No pKpn2146b/81715-82482

IS*Ecp1* (IS*1380* family), expected DR length = 5 bp

ttcgcaatagtaataCCTAG…ATGGGtaataaaagaggggc Yes^d^ chromosome/2746557-2744004 (898)

gctgaaggtctgggaCCTAG…TCAGAtgggaatatgatgct Yes^d^ chromosome/5405826-5409099 (1618)

aattccaactatttcCCTAG…TCAGGatttccttatttgct Yes^d^ pNDM-US/70431-74909 (2832)

ccgatatgaatcataCCTAG…CCAGGtcatattccttccgg Yes^d^ pKpn2146b/49709-46739 (1315)

IS*3000* (Tn*3* family), DR length measured here = 5 bp

taaaaggaacgttctGGGGT…ACCCCgttctcatggcaaga Yes pNDM-US/137590-140825

attcaatcacgttctGGGGT…ACCCCtttttgctgcgtcga No pKpn2146b/32882-27185

Tn*6187* (Tn*3* family), expected DR length = 5

catgatggactgataGGGGT…ACCCCcacatagagaccttc No pKpn2146b/64641-55334

IS*Kpn21* (IS*NCY* family), expected DR length = 5 bp

tatgcgtactcatgtTGTAA…TGACAcatgttgtagtagct Yes chromosome/2729639-2731916

tatgcgcacctgtatTGTAA…TGACAaacattgtcagcaca No pKpn2146c/48258-45981

IS*Ec22* (IS*66* family), expected DR length = 8 bp

tcagaaggactgtctGTAAG…CTTACgactgtcttgtcgct Yes pKpn2146c/54491-52038

tctggcaatttttcgGTAAG…CTTACatttttcgctcaatt Yes pKpn2146b/27331-29784

IS*903B* (IS*5* family), expected DR length = 9 bp

atccagtcccaaaatGGCTT…AAGCCtataaaagaaataaa No pKpn2146c/102929-101873

^a^ Oriented as in ISfinder; flank, lower case; terminal inverted repeat, upper case

^b^ DR observed (underlined), matching expected length

^c^ These IS*6100* copies are adjacent to integrons, a context in which DRs are rarely found.

^d^ IS*Ecp1* habitually transposes together with right-flanking DNA; coordinates and right end sequence are given for this entire transposed unit, as determined comparatively. Length in bp of the right-flanking co-transposed segment in parentheses.

**III. SUPPLEMENTARY FIGURES**

Fig. S1. Novel software applied in visualizing the last gap in the genome assembly. The script readStepper was run, inputting our Illumina read set and the seed sequence GTGGTCATTTTAATTGAGCT from the right end of the nearly-completed pKpn2146c. The algorithm trims off the seed and preceding sequence from matching reads, and sorts the remaining seed-extension sequences alphabetically. For illustration purposes, 50 representative lines were taken from the original 379-line output. Interpreting this image, the seed sequence is from a duplicated region whose two different flanks form two diagonal horns, under a zone of shared sequence. Further examination showed that one horn came from a chromosomal copy of the seed, and the other came from the pKpn2146c-closing copy. The demonstration of closure was completed by a second leftward run using the underlined seed sequence, which produced a single horn. Short arrows mark probable sequencing errors that produced reads disrupting the ideal two-horn shape. An unusual sequence source is illustrated here also, by the blue sequence which is from a free circular form of IS*Kpn21*.

Fig. S2. Scattered class 1 integron and integron-derived regions. Intact antibiotic-resistance cassette genes (blue): *ant(3´´)-Ib*, streptomycin resistance; *aac(6’)-Ib* and *aac(6´)-Ib-cr*, aminoglycoside resistance; *dfrA14*,trimethoprim resistance; *bla*_OXA1_, β-lactam resistance. The three *aac(6´)-Ib* alleles are distinguished by including in brackets the entry numbers from Table 1 of [5]. Slashes represent intrusions by insertion sequences, which appear to have inactivated a Δ*catB3* and Δ*ant(3´´)-Ib* gene. The shaded portion is an IS-truncated form of Tn*1331*. Other abbreviations: *attI1* and *attC*, cassette attachment sites; *intI1*, integrase gene; Δ, gene truncation.

Fig. S3. Plasmid pKpn2146a. Key, color coding of genes, mobile, unique regions and non-gene features. Inner ring, representative long matches to other plasmids.

Fig. S4. Free IS*Kpn21* circles. Upper panel. Two genome-integrated IS*Kpn21* copies and derived free circles, with counts of MiSeq read pairs supporting each form. Red, IS sequences; black, flanking genomic sequences; blue and green, direct repeat sequences. Lower panel. PCR tests. Primer pairs used in each lane are indicated. M, markers with sizes given at left.

Fig. S5. Support for rRNA operon assembly. Assembly of MiSeq reads outward from each end of the 16S and 23S rRNA sequences produced seven unique internal transcribed spacers with the indicated tRNA gene content, and eight unique flanks at each operon end. Solid lines indicate joints between these unique segments across the large rRNA genes, supported by the indicated number of PacBio reads (47 total), double arcs representing support from reads spanning the entire operon. Dashed lines denote two additional PacBio reads that clearly support joints that disagree with the rest of the data, suggesting recombination, perhaps in vivo in a culture subpopulation. Similar PacBio cross-joints were observed for one of 25 reads spanning the eight-copy group II intron.

Fig. S6. S.ma.I1 group II intron preference for promoters. Upper panel, exons of each intron copy in Kpn2146. Five of eight copies appear in promoters. “Element”, mobile DNA carrying the intron (the *waaU* site of copy B is not a known genetic element. “Uninterrupted”, accession exemplifying the uninterrupted form of the target site (only other interrupted examples could be found for copy G). Bottom panel, sequence logo of these eight integration sites.

**IV. SUPPLEMENTARY REFERENCES**

1. Carattoli A, Villa L, Poirel L, Bonnin RA, Nordmann P (2012) Evolution of IncA/C *bla*_CMY-2_-carrying plasmids by acquisition of the *bla*_NDM-1_ carbapenemase gene. Antimicrob Agents Chemother 56: 783-786.

2. Broberg CA, Palacios M, Miller VL (2013) Whole-genome draft sequences of three multidrug-resistant *Klebsiella pneumoniae* strains available from the American Type Culture Collection. Genome Announc 1: e00312-13.

3. Candales MA, Duong A, Hood KS, Li T, Neufeld RA, et al. (2012) Database for bacterial group II introns. Nucleic Acids Res 40: D187-190.

4. Siguier P, Perochon J, Lestrade L, Mahillon J, Chandler M (2006) ISfinder: the reference centre for bacterial insertion sequences. Nucleic Acids Res 34: D32-36.

5. Ramirez MS, Nikolaidis N, Tolmasky ME (2013) Rise and dissemination of aminoglycoside resistance: the *aac(6')-Ib* paradigm. Front Microbiol 4: 121.

6. Arakawa Y, Wacharotayankun R, Nagatsuka T, Ito H, Kato N, et al. (1995) Genomic organization of the *Klebsiella pneumoniae* *cps* region responsible for serotype K2 capsular polysaccharide synthesis in the virulent strain Chedid. J Bacteriol 177: 1788-1796.

7. Seo JH, Hong JS, Kim D, Cho BK, Huang TW, Tsai SF, Palsson BO, Charusanti P. 2012. Multiple-omic data analysis of *Klebsiella pneumoniae* MGH 78578 reveals its transcriptional architecture and regulatory features.BMC Genomics13: 679.

8. Burmann BM, Knauer SH, Sevostyanova A, Schweimer K, Mooney RA, et al. (2012) An α helix to β barrel domain switch transforms the transcription factor RfaH into a translation factor. Cell 150: 291-303.

9. Jensen SO, Reeves PR (2004) Deletion of the *Escherichia coli* O14:K7 O antigen gene cluster. Can J Microbiol 50: 299-302.
